# Synergistic dual-modified liposome improves targeting and therapeutic efficacy of bone metastasis from breast cancer

**DOI:** 10.1080/10717544.2017.1396384

**Published:** 2017-11-02

**Authors:** Xianzhu Ke, Wen Lin, Xiaokang Li, Hailiang Wang, Xin Xiao, Zheng Guo

**Affiliations:** a Department of Orthopedics, Xijing Hospital, Fourth Military Medical University, Xi'an, China;; b Department of Orthopedics, Hubei Cancer Hospital, Wuhan, China;; c Department of Clinical Laboratory, Huangshi Love & Health Hospital of Hubei Province, Huangshi, China

**Keywords:** Bone metastasis, Asp_8_ peptide, folate, bone targeting, dual-modified liposomes

## Abstract

Breast cancer frequently metastasizes to bone, where it leads to poor clinical prognosis. Due to the peculiarity of the bone microstructure, the uptake of drugs often happens at non-targeted sites, which produces a similar cytotoxicity in both cancerous and healthy cells. In this study, a rational strategy was implemented to take advantage of a combination of both an octapeptide with eight repeating sequences of aspartate (Asp_8_) and folate to create a more selective and efficient drug delivery system to target cancer cells in bone tissue. Asp_8_ and folate were conjugated to the distal ends of DSPE-PEG_2000_-maleimide and DSPE-PEG_2000_-amine to create DSPE-PEG_2000_-Asp_8_ and DSPE-PEG_2000_-Folate, respectively, which were incorporated onto the surface of a doxorubicin (DOX)-loaded liposomes (A/F-LS). Asp_8_, similar to the hydroxyapatite-binding domains of osteopontin and osteocalcin, has been used as bone-targeting moieties for exclusive delivery of drugs to bone. The folate moiety binds selectively to folate receptor-positive tumors. The dual-targeting effects were evaluated by both *in vitro* and *in vivo* experiments. By taking advantages of dual-targeting drug delivery, the dual-modified liposomal drug system could relieve pain and improve survival. Inspired by its enhanced therapeutic efficacy and low toxicity, DOX-loaded A/F-LS could serve as an effective drug system for targeted therapy of bone metastases.

## Introduction

Metastasis is the fatal stage in all cancers, which still remains incurable in spite of in depth research. Due to the peculiarity of the physiological environment, the skeleton is one of the most common organs to be affected by metastatic cancer. Among human cancers, breast cancer has the highest prevalence in bone metastasis (60–75% of breast metastases occur in bones) (Mundy, 2002). Tumor cells deposited in bone usually destroy the bone marrow microenvironment and stimulate osteoclast bone absorption, meanwhile, bone degradation further promotes tumor growth and metastases. Only 20% of patients with breast cancer are still alive five years after the diagnosis of bone metastases (Coleman, 2001). The clinical therapies for bone metastasis concentrate mainly on radiotherapy, surgical resection, and chemotherapy (Hao et al., 2016). However, the special pathological and physiological characteristics make bone metastases treatment very difficult. Since bone metastases usually possess multiple nodules, it is difficult to completely eliminate them by localized radiation therapy or surgical resection. For chemotherapy, because bones have low blood flow, systemic administration of drugs does not produce a sufficient drug concentration at the bone site (Jiang et al., 2012). To combat this issue, drugs are generally administered in high dosages and/or administered very frequently, both of which can lead to detrimental systemic side effects. Therefore, the management of bone metastases represents a huge clinical burden.

To meet this challenge, in chemotherapy researches, many attempts have focused on nanoparticle drug delivery systems (DDSs) (such as hydroxyapatite (HA) nanoparticles, liposomes, micelles) which could make a significant contribution to increase the permeability and retention (EPR) effect (Karacivi et al., 2017). However, because of a lack of specific targeting, it is difficult for the nanoparticles to accumulate in bone metastatic sites merely based on their nano-size. An ideal bone-targeting DDS for bone metastases should prolong blood circulation time with no aggregation, specifically accumulate in bone sites, and further target tumor cells. Additionally, it should exhibit optimized drug loading and releasing capabilities and biosafety (Vinay & KusumDevi, 2016). Thus, various targeted strategies have been applied in the field of nanocarriers to target metastatic sites in the bone. Among these strategies, an active targeted DDS is one of the most promising strategies (Chang et al., 2016). Hydroxyapatite is a unique mineral present in bone, which makes it an attractive target to develop bone-targeting DDS for bone-related pathologies (Jiang et al., 2012). Many molecules, such as bisphosphonates (BPs), exhibit specific HA binding feature. Among these molecules, a new approach employing peptides with aspartic acid repeating units for bone-targeting delivery seems promising. These amino acids exhibit a negative charge due to the carboxylate ligands, which can chelate calcium ions on the surface of HA (Addadi & Weiner, 1985). The use of amino acids as bone-targeting ligands has potential advantages compared with BPs. Unlike BPs, amino acids can be enzymatically degraded and cleared from the body after HA binding (Murphy et al., 2007), which minimizes the potential of cytotoxicity. Recently, Wang et al. developed liposomes modified with cyclic arginine-glycine-aspartic acid-tyrosine-lysine peptide (cRGDyk), which showed good therapeutic efficacy in a mice model of bone metastasis from prostate cancer (Wang et al., 2014). In recent years, an octapeptide with eight repeating sequences of aspartate (Asp_8_) have been used as bone-targeting moieties for the exclusive delivery of drugs to bone (Yokogawa et al., 2001). Hence, many groups have used the Asp_8_ as a bone-targeting ligand to deliver drugs to bone tissues for bone diseases therapy (Miller et al., 2008). Although single Asp_8_ could improve the bone-targeting delivery to some extent, the Asp_8_ modified nanocarriers may not be sufficient to target the tumor cells in bone. This is because the Asp_8_ was mainly targeted to the bone structure but not the tumor cells in it. Despite the effort invested up to this point, it is still a tremendous challenge to engineer the above-mentioned characteristics of an ideal bone-targeting DDS into a single ligand modified nanocarrier.

To address this need, the introduction of various biological ligands or antibodies into DDSs has provided the opportunity for the selective delivery of drugs to tumor cells. Such ligands can be recognized by specific receptors on certain types of cancer cell surfaces, which then induce the cellular uptake of the ligand-decorated nanocarriers via receptor-mediated endocytosis. Among cancer-specific ligands, folate, a small-molecule targeting ligand, has a high affinity (*K*_d_ ∼0.1–1 nmol/L) for the folate receptor (FR), which has been widely employed as a targeting moiety for various anticancer drugs and nanocarriers (Leamon & Reddy, 2004). FR is a 38 kDa glycosyl phosphatidylinositol-anchored protein over-expressed by many primary and metastatic cancers, including breast cancers, while its expression is highly restricted in normal cells (Weitman et al., 1992).

Doxorubicin (DOX) is an anthracycline antibiotic that possesses broad spectrum antineoplastic activity and is one of the most important anticancer agents in use (Hortobagyi, 1997). However, its clinical utility is hampered by dose-limiting cardiotoxicity, myelosuppression, and the developmental drug resistance (Arnold et al., 2004). To avoid such complications, the use of liposomes as nanocarriers for DOX has been explored in both animal and human trials (Mayer et al., 1989).

In this study, we designed a dual-targeting liposomal system modified with Asp_8_ and folate (abbreviated as A/F-LS), in which Asp_8_ could target the bone and folate could target the tumor cells in the bone. This dual-modified design is rarely reported in the bone tumor targeting study. Such novel dual-targeting concept was intended to improve the selective delivery of drugs to cancer cells and to reduce intrinsic toxicity to healthy cells beyond the reliance upon the EPR effect and monotarget modification.

The resultant liposomes were characterized and their targeting efficiency was studied both *in vitro* and *in vivo* using fluorescent probe. Further to this, enhanced therapeutic efficacy *in vivo* of A/F-LS containing DOX including pain relief and overall survival improvement was also observed. The findings have provided valuable preclinical data to validate a noninvasive, efficient targeted ligand-nanotherapy for the treatment of bone metastasis from breast cancer.

## Experimental materials

### Materials

Hydrogenated soy phosphatidylcholine (HSPC) and cholesterol (Chol) were purchased from Lipoid GmbH (Mannheim, Germany). 1,2-Distearoyl-sn-glycero-3-phosphoethanolamine-N-methoxy (polyethyleneglycol) (ammonium salt) (DSPE-mPEG_2000_), 1,2-distearoyl-snglycero-3-phosphoethanolamine-N-polyethyleneglycol_2000_-amine (DSPE-PEG_2000_-NH_2_), and DSPE-PEG_2000_-maleimide (DSPE-PEG_2000_-Mal) were purchased from Avanti Polar Lipids, Inc. (Alabaster, AL). Asp_8_ (CDDDDDDDD) and folate were synthesized by Top-peptide Biotechnology (Shanghai, China). Doxorubicin hydrochloride was supplied by Haizheng Co. (Zhejiang, China). 4,6-Diamidino-2-phenylindole (DAPI) was purchased from Beyotime (Haimen, China). All chemicals were of reagent grade and were obtained from Sigma-Aldrich (St. Louis, MO), unless otherwise stated.

MDA-MB-231 human mammary adenocarcinoma cell lines purchased from the Cell Resource Centre of IBMS (Beijing, China) were maintained in culture medium consisting of Dulbecco’s modified eagle’s medium (DMEM) supplemented with 10% FBS, 100 IU/mL penicillin, and 100 mg/mL streptomycin. The cells were maintained in a 37 °C humidified incubator in a 5% CO_2_ atmosphere.

Athymic female nude mice (5–6 weeks) and female Sprague-Dawley (SD) rats (180–220 g) were purchased from Animal Laboratory Center of Hubei Cancer Hospital (Wuhan, China). All animals were handled according to the code of ethics in research, training and testing of drugs as laid down by the Animal Care and Use Ethics Committee of Hubei Cancer Hospital.

## Methods

### Synthesis and characterization of targeting molecule conjugates

The synthesis of folate-NHS followed the procedure previously reported by Xiang et al. (2013). Asp_8_ was conjugated with DSPE-PEG_2000_-Mal (1:1 molar ratio) and folate-NHS was conjugated with DSPE-PEG_2000_-NH_2_ (1:1 molar ratio) in chloroform that contained triethylamine (TEA, 5 eq.) at room temperature for 24 h while stirring, respectively. The reaction mixture was dialyzed (molecular weight cutoff (MWCO) 3.5 kDa) in distilled water for 48 h to remove the chloroform and unreacted peptides. The final solution was evaporated by rotary evaporation and stored at –20 °C for further use. The existence of the conjugations was confirmed with MALDI-TOF mass spectrometry (MALDI-TOF MS).

### Preparation of liposomes

The non-modified liposome (N-LS) with composition of HSPC:Chol:DSPE-PEG_2000_ (mol/mol, 50:36:14), was prepared by direct hydration of a lipid film as described previously (Yang et al., 2015), with minor modifications. Briefly, all lipids or hydrophobic probe (Cy5.5) were dissolved with chloroform–methanol (3:1, v/v) in a pear-shaped flask and were subsequently evaporated to form dry film using a rotary evaporator under vacuum. The lipid film was then hydrated using 300 mM citric acid buffer solution at 50 °C for 30 min. To control the size, the lipid dispersion was extruded 11 times through 100 nm polycarbonate filters using a mini extruder (Avanti, Calgary, Canada). The Asp_8_-modified liposomes (A-LS) and Folate-modified liposomes (F-LS) were prepared by following the same procedures, except the DSPE-PEG_2000_ was partially substituted by DSPE-PEG_2000_-Asp_8_ and DSPE-PEG_2000_-Folate conjugates (1%, 5%, 10%, and 15%, molar ratio), respectively. For Asp_8_/Folate-co-modified liposomes (A/F-LS), the content of DSPE-PEG_2000_-Asp_8_ and DSPE-PEG_2000_-Folate was 15% and 10%, respectively.

DOX was loaded into various liposomal formulations using the pH gradient method at 1:20 drug/lipids mass ratio as described by a previous report (Li et al., 2016). Briefly, liposome suspension was directly added into 300 mM citric acid buffer drug solution, and then adjusted the pH of extrinsic phase to 7.5 using Na_2_CO_3_ solution, then incubated for about 30 min. Finally, the DOX-loaded liposomes were filtered and sterilized by a 100 nm polycarbonate filter and subpackaged to aseptic ampoules.

### Characterization of liposomes

The mean diameter and zeta-potential of each formulation were determined in three serial measurements using a Malvern Zetasizer Nano ZS90 instrument (Malvern Instruments Ltd., Malvern, UK). The morphology of A/F-LS was observed via transmission electron microscopy (TEM, HITACHI, H-7650, Tokyo, Japan). The DOX encapsulation efficiency (EE) of each formulation was determined by an HPLC as described by a previous report (Li et al., 2016). The *in vitro* stability of liposomes in 10% FBS was evaluated using a Turbiscan Lab^®^ Expert (Formulaction, Toulouse, France), an innovative analytical instrument able to determine the small changes of colloidal systems. A/F-LS was diluted by cell culture medium (90% DMEM) containing 10% FBS and analysis for 48 h. Measurements were carried out using a pulsed near infrared LED at a wavelength of 880 nm.

### *In vitro* drug release study

The dialysis method was used to study the *in vitro* release of drug from the various formulations of liposomes. The release medium was phosphate buffer solution (PBS, pH 7.4) containing 0.5% Tween-80 (w/w). Briefly, 0.5 mL of liposome dispersion was transferred to a dialysis bag (MWCO 12,000-14,000 Da) and dialyzed against 50 mL of the medium with continuous gentle stirring for 24 h at 37 °C. At various time points, 800-μL aliquots were withdrawn from the conical flask for drug analysis and an equal volume of the medium was added. The leakage of DOX was determined using HPLC as previously described (Li et al., 2016).

### Optimization of ligand density of liposomes

#### HA-binding assay

To investigate the effect of Asp_8_ density on HA-binding, DOX-loaded A-LS was prepared at different peptide densities (1%, 5%, 10%, and 15%, molar ratio). Briefly, HA beads were suspended in Tris/HCl solution (50 mM Tris/HCl and 150 mM NaCl, pH 7.4) at 15 mg/mL. Liposomes (600 μL) were mixed with 300 μL of HA suspension or 300 μL of the Tris/HCl solution as a control, followed by gentle shaking for 1 h at room temperature. After centrifugation at 10,000×*g* for 5 min, the HA precipitate was separated from the unbound liposomes in the supernatant. Unbound liposomes were quantified through spectrophotometry to measure DOX encapsulated in the liposome. The degree of HA binding was calculated according to the following formula (Zhang et al., 2017):

HA binding (%)=(A-B)/B×100%

where *A* is the amount of DOX in the control group and *B* is the amount of DOX in the experimental groups.

#### Effect of folate density on cellular uptake of liposomes

To investigate the effect of folate density on cellular uptake, DOX-loaded F-LS was prepared at different folate densities (1%, 5%, 10%, and 15%, molar ratio). MDA-MB-231 cells were seeded at a concentration of 5 × 10^5^ cells/well in six-well plates for 24 h. Then, the cells were incubated with different liposomal formulations for 2 h at 37 °C and the cells were rinsed with cold PBS, trypsinized and washed three times with cold PBS. The samples were then centrifuged and resuspended with PBS. Approximately, 10^5^ cells were applied immediately using a flow cytometry (FCM) (BD FACSCalibur, San Jose, CA).

### *In vitro* cytotoxicity assay

Cytotoxicity of free DOX and various liposomal formulations containing DOX against MDA-MB-231 cells were evaluated with MTT assay. The cells were seeded into a 96-well plate at a density of approximately 4000 cells/well. Then, cells were treated with various formulations at a range of concentrations. After the cells were further incubated for 72 h, 20 μL of MTT solution (5 mg/mL in PBS) was added to each well. After 4 h incubation, the percentage of cell viability was determined on the basis of absorbance at 490 nm by a plate reader (Model 680, BIO-RAD, Hercules, CA).

### Pharmacokinetic studies

Sixteen SD rats were randomly divided into four groups and intravenously administrated via the tail vein with free DOX, DOX-loaded A-LS, DOX-loaded F-LS, and DOX-loaded A/F-LS at an equivalent dose of 5 mg/kg DOX, respectively. Blood samples were drawn from the carotid vein at 0.083, 0.25, 0.5, 1, 2, 3, 4, 6, 8, 12, and 24 h post-injection. The plasma samples were collected following centrifugation at 3000 rpm for 10 min and stored at –20 °C until assays.

To prepare samples for analysis, 100 mL methanol containing 60 ng/mL daunorubicin (internal standard) was added into 50 mL plasma to precipitate the proteins. The mixture was vortexed and subsequently centrifuged at 12,000 rpm for 10 min with the supernatant mixed with an equal volume of deionized water and subjected to an LC–MS/MS analysis as described previously (Gong et al., 2011).

### Animal model of bone metastasis

A mice model of bone metastasis from breast cancer was established by intra-tibia injection of MDA-MB-231 cells, as previously described (Thamake et al., 2012). Briefly, after anesthetized by intra-peritoneal injection of chloral hydrate, the tibia of right hind limb of nude mice was carefully exposed, and a 23-gauge needle was inserted into the intramedullary canal of the bone, followed by injection of 20 μL of MDA-MB-231 cells (1 × 10^6^). Then, the injection site was sealed with bone wax and the wound was finally closed. The mice were placed in animal room and were given free access to food and water throughout the experiment.

### *In vivo* imaging

Cy5.5-loaded liposomes were prepared for bone-targeting evaluation. Eight days after surgery, the mice bearing MDA-MB-231 tumors were administered via tail vein injection with Cy5.5-loaded different liposomal formulations. Six hours after the injection, the *in vivo* imaging was performed with an IVIS Lumina XR (Caliper Life Sciences Inc., Hopkinton, MA).

### Pain behavior evaluation

Tumor-bearing mice were randomly divided into six groups (six mice per group) intravenously receiving one of the following samples via the tail vein at eight day following tumor cells injection: free DOX, DOX-loaded N-LS, DOX-loaded A-LS, DOX-loaded F-LS, DOX-loaded A/F-LS, and physiological saline group (5 mg/kg) at a single DOX dose of 5 mg/kg body weight. The pain-related behaviors of the mice were tested at 4, 6, 8, 10, 12, 14, 16, 18, 20, 22, and 24 days following tumor cells injection. After a five-minute acclimation period, the spontaneous lifting time and the number of flinches were measured over a four-minute observation period according to a previous report (Vermeirsch et al., 2004). Every lift of right hind limb not related to walking or grooming was considered to be one flinch, and the duration of the lift was counted until the paw again touched the walking surface.

### *In vivo* anti-tumor effect

The mice bearing MDA-MB-231 tumors were randomly divided into the following six groups (six mice per group): free DOX, DOX-loaded N-LS, DOX-loaded A-LS, DOX-loaded F-LS, DOX-loaded A/F-LS, and physiological saline group. Eight days after cell injections, each mouse received a dose of 5 mg/kg four times every two days. The mice in each group were used to monitor survival. The survival time was calculated from day 0 (tumor inoculation) to the day of death. Kaplan–Meier’s survival curves were plotted for each group. Meanwhile, the body weight of each mouse was measured every two days.

### Safety assay

In order to study the toxicity of DOX-loaded A/F-LS, the mice were administered with free DOX and DOX-loaded A/F-LS at a single DOX dose of 5 mg/kg body weight via the tail vein, respectively. The mice treated with PBS were investigated as a control group. At day 18 after the treatment, the heart, liver, spleen, and kidney were harvested and embedded into paraffin, sectioned at a thickness of 5 μm, and stained with hematoxylin and eosin (H&E) for histology study. Meanwhile, hemogram analysis was performed to further evaluate the *in vivo* safety properties of the nanocarriers.

### Statistical analysis

The data are presented as the means ± standard deviation (SD). The difference between any two groups was determined via ANOVA. *p* < .05 was considered to be statistically significant.

## Results and discussion

### Synthesis of functional conjugates

The A/F-LS was developed by the modification of two synthesized functional materials, DSPE-PEG_2000_-Asp_8_ and DSPE-PEG_2000_-Folate. These functional materials were synthesized as shown in Figure S1. The Asp_8_ was terminated with cysteine to introduce free sulfhydryl (–SH), and then conjugated to DSPE-PEG_2000_-Mal via the sulfhydryl-maleimide reaction, which enabled Asp_8_ to be conjugated at a specific site (–SH) (Figure S1(A)). To obtain the DSPE-PEG_2000_-Folate, our approach involved carbodiimide-mediated coupling of folate to readily attainable DSPE-PEG_2000_-NH_2_ (Figure S1(B)). The MALDI-TOF MS results confirmed the successful formation of DSPE-PEG_2000_-Asp_8_ and DSPE-PEG_2000_-Folate, with the observed mass–charge ratios of approximately 3864.154 (Figure S1(C), marked by an arrow) and 3239.984 (Figure S1(D), marked by arrow), which was equal to the theoretical mass–charge ratios of 3864 and 3239, respectively. The final product was then used for preparing targeted liposomes in experiments.

### Characterization of liposomes

The physico-chemical properties of the four distinct liposome formulations are summarized in Table 1. The DOX EE of all liposomes was more than 90%, and the modifications of Asp_8_ and/or Folate on the surfaces of the liposomes did not affect the ultimate EE. For DDS, nanoparticle size would be a precondition and a crucial factor which decided the fate of DDS both *in vivo* and *in vitro*. After EE study, the particle sizes of various liposomes were further analyzed by laser particle analyzer. As shown in Table 1, the sizes of the N-LS, A-LS, F-LS, and A/F-LS were between approximately 95.82 ± 0.13 nm and 98.61 ± 0.11 nm. It can be concluded that the sizes of the N-LS, A-LS, F-LS, and A/F-LS were not significantly affected by the Asp_8_ or Folate modification. As shown in Figure S2(A), TEM images of A/F-LS demonstrated that the particle sizes were similar to those determined using a laser particle analyzer (Figure S2(B)).

**Table 1.** Characteristics of the liposomes.

Sample ID	Liposomes components (mol ratio of total lipid)	Diameter (nm)	Polydispersity index	EE (%)
N-LS	HSPC/Chol/DSPE-PEG_2000_ (50:36:14)	95.82 ± 0.13	0.09 ± 0.01	93.4 ± 1.9
A-LS	HSPC/Chol/DSPE-PEG_2000_/DSPE-PEG_2000_-Asp_8_ (50:36:4:10)	96.21 ± 0.12	0.10 ± 0.02	91.7 ± 1.2
F-LS	HSPC/Chol/DSPE-PEG_2000_/DSPE-PEG_2000_-Folate (50:36:13:1)	96.14 ± 0.15	0.09 ± 0.01	94.5 ± 1.8
A/F-LS	HSPC/Chol/DSPE-PEG_2000_/DSPE-PEG_2000_-Asp_8_/DSPE-PEG_2000_-Folate (50:36:3:10:1)	96.61 ± 0.11	0.11 ± 0.01	92.2 ± 2.1

The data are expressed as the mean ± SD for three different preparations (*n* = 3).

The *in vitro* DOX release study was also performed to examine the drug release property of the liposomes. As shown in Figure S2(C), there were no pronounced differences in DOX release behavior among the four types of liposomes at each time point. The similar physicochemical characteristics of liposome allowed us to specifically compare the effects of ligands modification on the liposome uptake and anticancer abilities.

The A/F-LS stability against physiological conditions is a prerequisite for further application *in vivo*, and thus, 10% FBS in PBS was employed to mimic the *in vivo* situation. The stability of the A/F-LS in the 10% FBS was evaluated using Turbiscan Lab^®^ Expert. According to this judgment (Celia et al., 2009), the transmission or back-scattering profiles (less than 0.5%) obtained (Figure S2(D)) indicate there was no apparent aggregation or sedimentation occurred of A/F-LS in the culture medium during 48 h.

### Optimization of ligand density of liposomes

**Figure 1.**
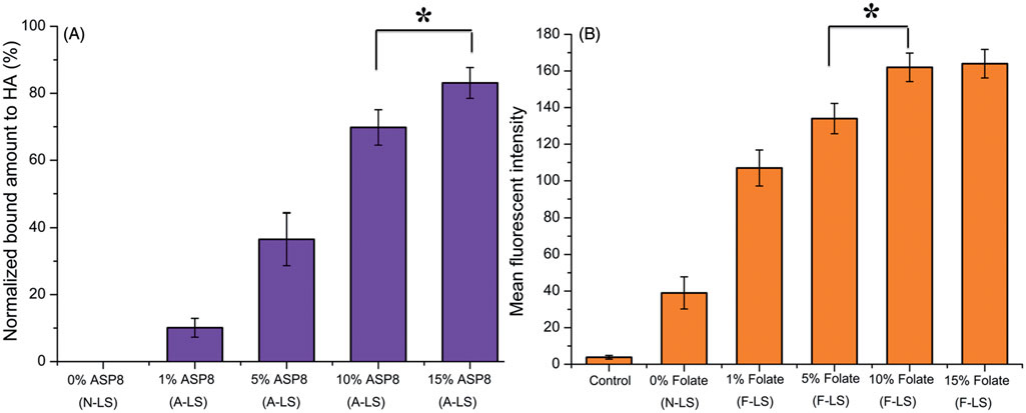
Optimization of ligand density of liposomes. HA-binding ratio of DOX-loaded liposomes with different densities of DSPE-PEG_2000_-Asp_8_ (A). Cellular uptake of DOX-loaded liposomes with different densities of DSPE-PEG_2000_-Folate (B) in MDA-MB-231 cells after incubation for 2 h at 37 °C (*n* = 3). *p < .05.

The density of the DSPE-PEG_2000_-Asp_8_ on the surface of the liposomes proved to be a key factor influencing the bone-targeting capacity of A/F-LS. In this section, the binding efficiencies of different densities of Asp_8_ in liposomes to HA were studied. The data presented in Figure 1(A) clearly demonstrated the capability of Asp_8_ modified liposomes (A-LS) to bind to HA. The binding quantity increased markedly as the Asp_8_/lipid molar ratio in the liposome formulation increased. Binding of liposome to HA was impacted by the zeta potential and interfacial tension, etc. through the steric, electrostatic, and chelating effects involved in the binding mechanism (Neves et al., 2002). As described above, increase of the Asp_8_/lipid molar ratio enhanced the net negative charge on the liposome surface, which might be responsible for the increased HA binding. Considering the above results, the molar ratio of 15% for Asp_8_/lipid was selected in the next experiments.

As the density of folate density in liposomes was an important factor that will influence the tumor-targeting efficiency of A/F-LS greatly, the cellular uptake of liposomes with modifications of different densities of folate was evaluated in MDA-MB-231 cells to guide the formulation optimizing process. It was reported that FRs were over expressed in MDA-MB-231 cells (Trombino et al., 2013). As shown in Figure 1(B), when the folate/lipid molar ratio was 1%, F-LS showed no significant increase of binding compared with N-LS (*p* > .05). While the cellular uptake of A-LS was significantly influenced by the increase of folate/lipid molar ratio from 5% to 10%. With the further increase of the ratio to 15%, there was no remarkable difference in cellular uptake compared with the F-LS with a 10% ratio likely due to saturation of the FR on the surface of cells. Limited by the number of receptors and the recycling of endocytosis, receptor mediated endocytosis is a saturated pathway, which restricts the amount of the nanocarriers that are available for cellular uptake. Accordingly, the optimal density of DSPE-PEG_2000_-Asp_8_ and DSPE-PEG_2000_-Folate on the liposomes was chosen to be 15% and 10% (molar ratio), respectively. It should be noted that the formulation screening results here may not be the optimal results, but this will not influence the final conclusion found in this paper.

### Cytotoxicity

**Figure 2.**
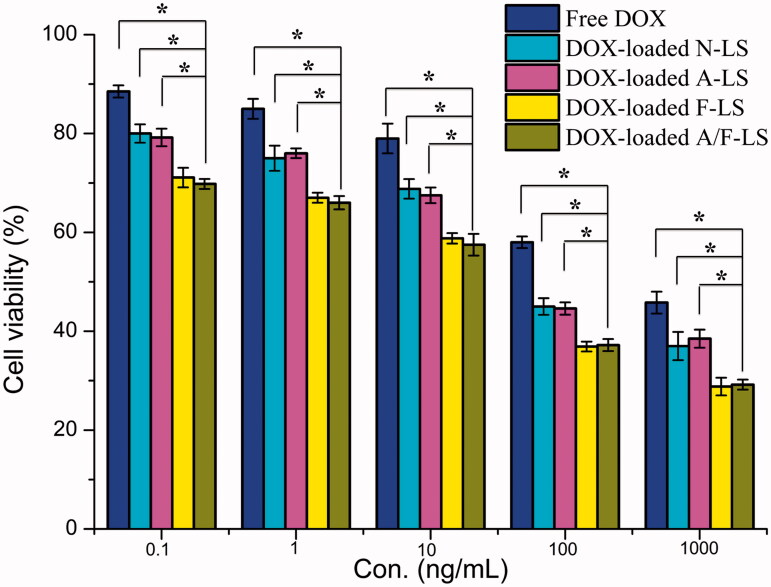
The cytotoxicity of free DOX and various liposomes containing DOX (*n* = 3). *p < .05.

MTT assay was conducted to evaluate the *in vitro* cytotoxicity of various liposomal formulations containing DOX in MDA-MB-231 cells (FR-positive cell models). As shown in Figure 2, free DOX could result in obvious anti-proliferative effects to MDA-MB-231 cells in a concentration-dependent manner, thus proving the anticancer effect on such kind of tumors. Among these DOX-loaded liposomes, the improved cellular uptake led to an anticipated enhanced anti-proliferation effect. This showed that the folate modified liposomes (DOX-loaded F-LS and DOX-loaded A/F-LS) significantly (*p* < .05) increased the cytotoxicity, with an IC_50_ of 17.8 μg/mL for DOX-loaded F-LS and 15.3 μg/mL for DOX-loaded A/F-LS, compared to 100.5 μg/mL for DOX-loaded A-LS and 94.9 μg/mL for DOX-loaded N-LS, respectively. The cytotoxicity studies demonstrated that the effect of folate on the modified liposomes promoted anti-proliferative activities in MDA-MB-231 cells that over-expressed FRs. In addition, we found that all liposomes demonstrated stronger anti-proliferative activities in the cells compared to free DOX indicating the enhanced endocytic uptake of nanocarriers by tumor cells.

### Pharmacokinetic studies

The mean plasma concentration–time profiles of the formulations after intravenous administration to SD rats are illustrated in Figure S3. It was found that dual-modified liposomes (A/F-LS) and mono-modified liposomes (A-LS or F-LS) showed similar blood concentration–time curves. However, the modified liposomes displayed different pharmacokinetic parameters with the N-LSs. There may be some receptors or receptor-like materials in the rat plasma, which can reversibly bind with the folate or Asp_8_ in the surfaces of the modified liposomes. Therefore, the targeted liposomes might be temporarily restored in the plasma and slowly dissociate from the contents in plasma, and this consequently lead to the superior circulation time of modified liposomes in rats.

Both the dual-modified liposomes and mono-modified liposomes showed initial high blood circulating levels, while N-LSs and free DOX were quickly cleared from the systemic circulation. The pharmacokinetic parameters are presented in Table 2. A/F-LS demonstrated significantly slower clearance rate (CL) and higher AUC when compared with N-LS. Furthermore, no significant difference in CL and AUC was observed between the dual-modified liposomes and mono-modified liposomes. It suggested that the conjugation of an adequate amount of Asp_8_ and folate on the surface of liposomes did not impair the long-circulation characteristic of PEG. Overall, A/F-LS possess a desirable pharmacokinetic profile, making it suitable for *in vivo* targeting drug delivery via systemic administration.

**Table 2.** Pharmacokinetic parameters of DOX following intravenous administration of free DOX and various DOX-loaded liposomes (*n* = 6).

Parameters	Free DOX	DOX-loaded N-LS	DOX-loaded A-LS	DOX-loaded F-LS	DOX-loaded A/F-LS
AUC_(0–_*_*t*_*_)_ (mg/L/h)	3.399 ± 0.145*	9.392 ± 1.502*	43.812 ± 7.403	39.005 ± 7.093	43.874 ± 6.812
*t*_1/2_ (h)	2.897 ± 0.654*	4.621 ± 0.796	6.434 ± 0.511	6.024 ± 0.449	7.087 ± 0.402
*k* (h^–1^)	0.241 ± 0.054*	0.112 ± 0.010	0.101 ± 0.003	0.122 ± 0.012	0.112 ± 0.011
CL (L/h/kg)	1.498 ± 0.061*	0.528 ± 0.101*	0.114 ± 0.009	0.118 ± 0.021	0.114 ± 0.009

* *p* < .05 significantly different with that of DOX-loaded A/F-LS.

### *In vivo* imaging

**Figure 3.**
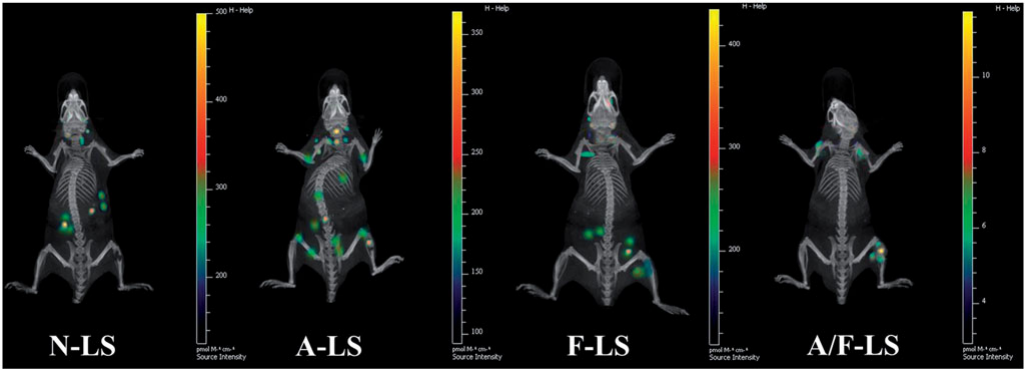
Biodistribution of Cy5.5 contained in various liposomes in mice bearing MDA-MB-231 tumors determined by an IVIS^®^ Spectrum-CT.

The selective distribution of drug formulated nanocarrier in tumors could potentially benefit the antitumor activity of chemotherapy *in vivo*. To verify this, *in vivo* tumor targeting efficiency of liposomal DDS in a bone metastasis model was evaluated and monitored in real time by *in vivo* NIR fluorescence imaging using Cy5.5-loaded various liposomes. Figure 3 shows representative fluorescent images overlapped with X-ray images of these mice. Based on whole body imaging, the right hind limb of the mice accumulation was much higher for the A-LS and A/F-LS group. While, there were no signals in the bone of animals treated with N-LS. These initial data provided substantial evidence that Asp_8_ functionalized liposomes (A-LS and A/F-LS) exhibited good bone-targeting ability *in vivo*. More importantly, the accumulation of A/F-LS in the tumor sites in the bone is higher than that of A-LS. This phenomenon indicted that the introduction of Asp_8_-induced bone-targeting in liposomes, and the incorporation of folate further enhanced its accumulation in tumors.

These initial data, resulting from *in vivo* imaging, provided substantial evidences that A/F-LS has the potential to accomplish specific targeting of drug to the metastasized tumor in the bone *in vivo*.

### *In vivo* therapeutic efficacy

**Figure 4.**
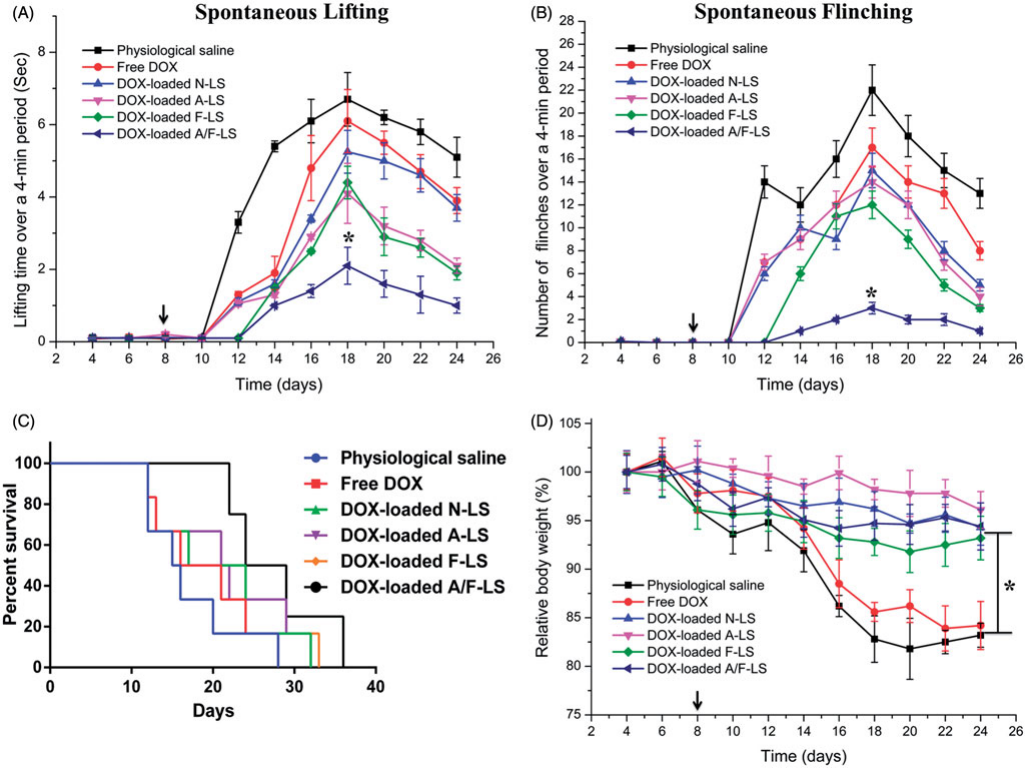
*In vivo* therapeutic efficacy evaluation. Spontaneous time lifting of tumor-bearing right bind limb over a four-minute observation period (A). Number of flinches of tumor-bearing right hind limb over a four-minute observation period (B). Kaplan–Meier’s survival curves (C). Body weight changes in tumor-bearing mice after treatments with various samples (D) (*n* = 6). *p < .05.

The clinical therapeutic benefits are mainly determined based on the quality of life and prolonged survival time of cancer patients. Bone pain is a frequent symptom of bone metastases. The mechanisms responsible for bone pain are poorly understood but seem to be a consequence of tumor-induced bone lesion (Currie et al., 2013). Thus, pain behavior of tumor-bearing mice treated with different formulations at day 8 post-injection of MDA-MB-231 cells was measured to evaluate therapeutic efficacy of liposomal DDS. As shown in Figure 4(A), the lifting time gradually increased over time reaching an maximal level at day 18, which is 6.7 ± 0.7 s for physiological saline group, 6.1 ± 0.9 s for free DOX group, 5.3 ± 0.6 s for DOX-loaded N-LS group, 4.1 ± 0.8 s for DOX-loaded A-LS group, 4.4 ± 0.5 s for DOX-loaded F-LS group, and 2.1 ± 0.5 s for DOX-loaded A/F-LS group. Although DOX-loaded A-LS group and DOX-loaded F-LS group can relieve pain compared with physiological saline group, there is no significant difference between them. Comparatively, lifting time of DOX-loaded A/F-LS group was significantly lower than the other groups (*p*<.05). The number of flinches behavior was observed to further evaluate pain behavior (Figure 4(B)). Consistent with lifting time results, the number of flinches also gradually increased over time, reached a maximal level at the day 18. The result is 22.23 ± 2.45 for physiological saline group, 17.02 ± 1.62 for free DOX group, 15 ± 1.43 for DOX-loaded N-LS group, 14.39 ± 1.58 for DOX-loaded A-LS group, and 12.08 ± 0.96 for DOX-loaded F-LS group. As we expected, DOX-loaded A/F-LS treatment can significantly attenuate flinching (3.25 ± 0.63), showing statistic difference compared with other groups.

In further investigation of the potential of various formulations in anti-metastasized tumor therapy *in vivo*, the Kaplan–Meier survival curve of intraosseous MDA-MB-231-bearing mice was used (Figure 4(C)). Pain behavior of the mice reflects their survival time. As expected, treatment with DOX-loaded A/F-LS significantly prolonged the median survival time (27 days), which was 1.7, 1.4, 1.2, and 1.3-fold higher than that of physiological saline, free DOX, DOX-loaded A-LS, and DOX-loaded F-LS, respectively. As shown in Figure 4(C), it can be seen that although DOX-loaded F-LS treatment can relief pain and delay death, it has no significant effects on the mean survival time. These results revealed the restricted therapeutic effects of DOX as chemotherapeutic agent, due to F-LS’s intrinsic drawbacks in treating bone metastases. For DOX-loaded A-LS, although it is well documented that the nanocarrier can aggressively favor the accumulation of drug in the bone via bone-targeting ligands (Figures 1(A) and 3), it is still not effective in targeting to tumor cells. In contrast, DOX-loaded A/F-LS take the advantages of dual-targeting drug delivery, contributing to the enhanced therapeutic efficacy of bone metastasis.

Meanwhile, the body weight variation of mice was monitored during the experimental period. As shown in Figure 4(D), more than 15% of weight loss was found in the free DOX group at the end of experiment. The weight loss of free drug groups was likely due to the non-targeted characteristics and tumor cachexia. While, the much smaller of weight loss of mice in DOX-loaded A/F-LS group than that of free DOX group during the whole experimental period, indicated the dual-modified modified liposomes could reduce unspecific cellular uptake through bone-targeted delivery.

### Toxicity studies

**Figure 5.**
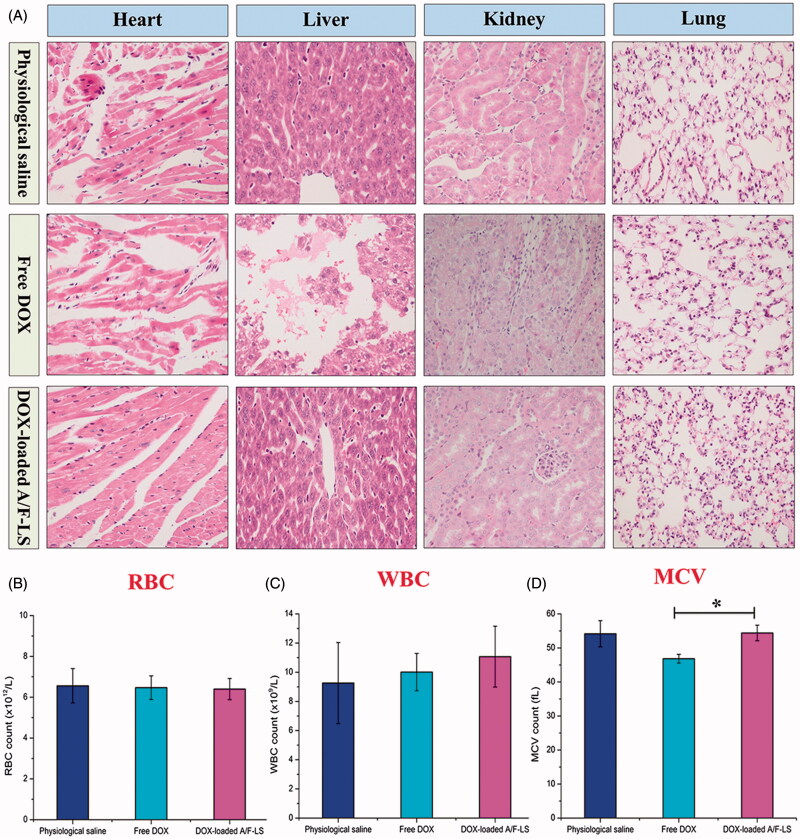
*In vivo* safety evaluation. HE staining of major organ sections from mice receiving different formulations on the day 18 (A). Hematological indicators of RBC (B), WBC (C), and MCV (D) on day 18 after inoculation (*n* = 3). *p < .05.

The goal for a DDS is to achieve optimal therapeutic efficacy with acceptable safety profiles during *in vivo* applications. With respect to the safety evaluation, the histology and hemogram analysis were performed during the experimental period. As shown in Figure 5(A), free DOX displayed histological damages in the organs of heart, liver and kidney, while the DOX-loaded A/F-LS only displayed mild liver toxicity. As shown in Figure 5(B–D), there was no obvious decrease in the red blood cell (RBC) and white blood cell (WBC) levels in free DOX and DOX-loaded A/F-LS. In the free DOX group, the mean corpuscular volume (MCV) was significantly decreased. Overall, these results demonstrated that DOX-loaded A/F-LS largely alleviated the toxicity of DOX from histological and hematological indicators and was relatively safe at the tested dose.

## Conclusions

In this study, Asp_8_ and folate conjugated liposomal DDS was designed for the treating bone metastasis in a mice model. A series of tests, including an *in vitro* HA-binding assay and *in vivo* distribution imaging, indicated that A/F-LS has a strong bone targeting effect. The high cellular uptake of A/F-LS by FR-rich tumor cells was confirmed by FCM, which resulted in high cytotoxicity of loaded DOX. Pharmacokinetic study and tissue distribution suggested that the dual-modified liposomes had prolonged blood circulation time, which favored accumulation of loaded DOX in the tumor. Noticeably, DOX-loaded A/F-LS showed enhanced therapeutic efficacy in treating bone metastasis including pain relief and overall survival improvement, by taking advantages of targeted drug delivery. Although preliminary, our study demonstrated a new avenue for treatment and experimental investigation of bone metastasis and encourages further studies on the application of the dual-targeted liposomes as an efficient delivery system of therapeutic agents in bone metastasis chemotherapy.

## Supplementary Material

IDRD_Guo_et_al_Supplemental_Content.doc
