# Supplementary material for: Synergistic dual-modified liposome improves targeting and therapeutic efficacy of bone metastasis from breast cancer
Source: Drug Deliv. 2017 Nov 2;24(1):1680–9. doi: 10.1080/10717544.2017.1396384 (PMC8241154; doi:10.1080/10717544.2017.1396384)
Supplement: IDRD_Guo_et_al_Supplemental_Content.doc [file IDRD_A_1396384_SM7498.doc]

**Supplementary information**


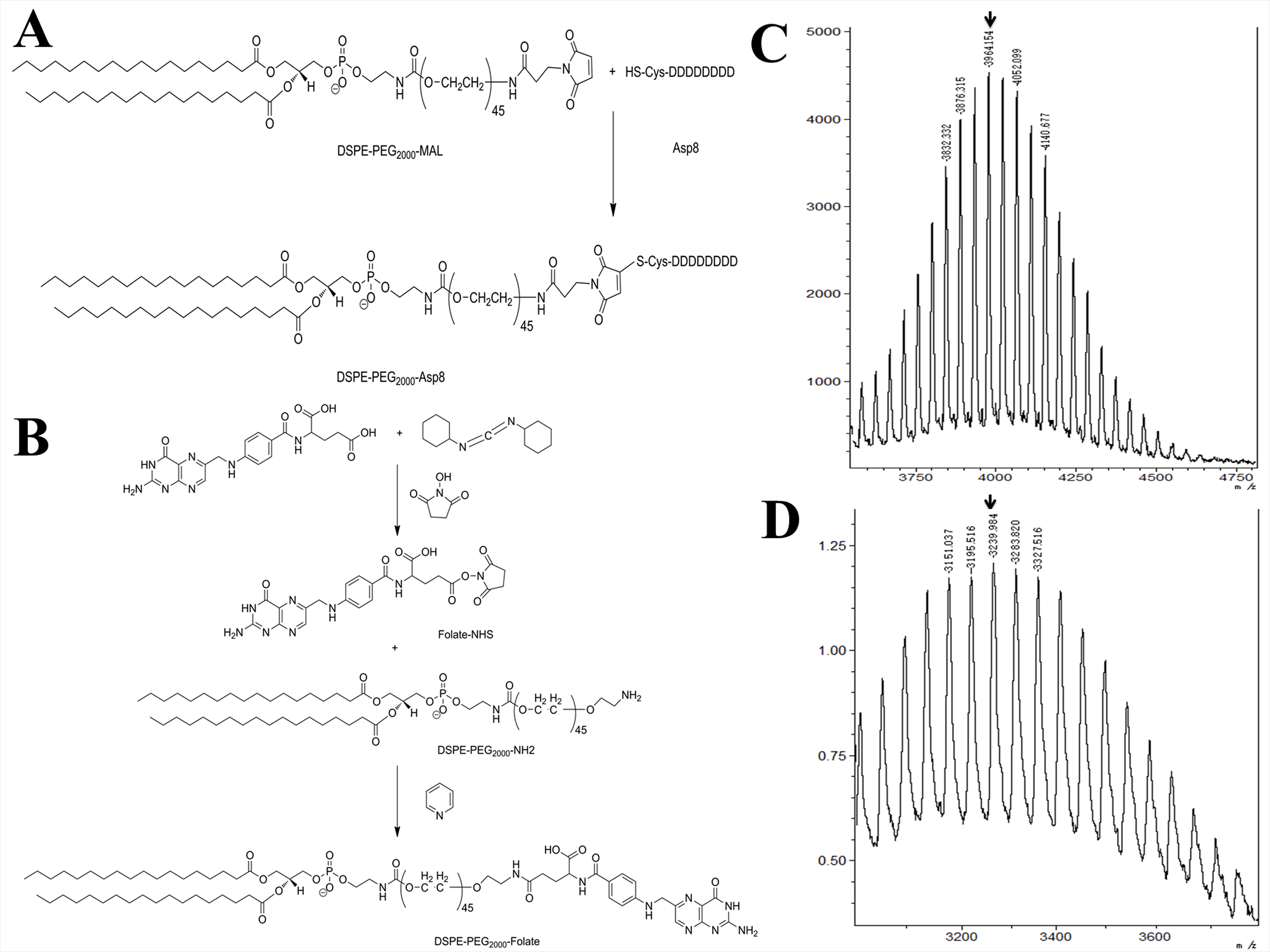


**Figure S1.** Principle of the synthesis of DSPE-PEG_2000_-Asp_8_ (A) and DSPE-PEG_2000_-Folate (B). MALDI-TOF mass spectra of DSPE-PEG_2000_-Asp_8_ (C) and DSPE-PEG_2000_- Folate (D).

**
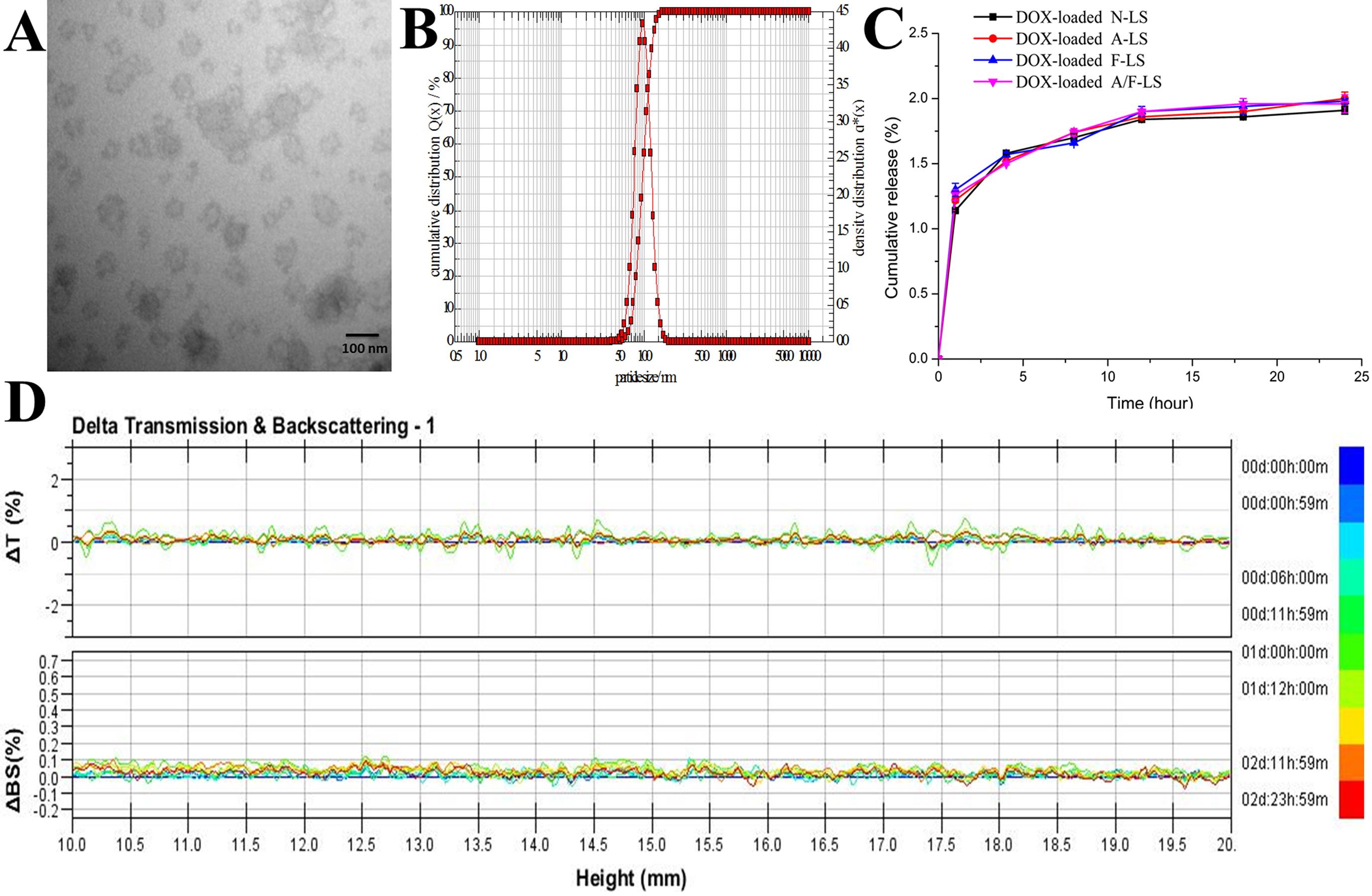
**

**Figure S2.** Physicochemical characterization of A/F-LS containing DOX. Morphological appearance of DOX-loaded A/F-LS based on TEM (A). Particle size distribution of DOX-loaded A/F-LS (B). *In vitro* release of DOX from various liposomes at 37 °C (C). Stability of DOX-loaded A/F-LS in the presence of 10% FBS. The transmission and backscattering profiles were measured at each time point using a Turbiscan Lab® Expert analyser (D).

**
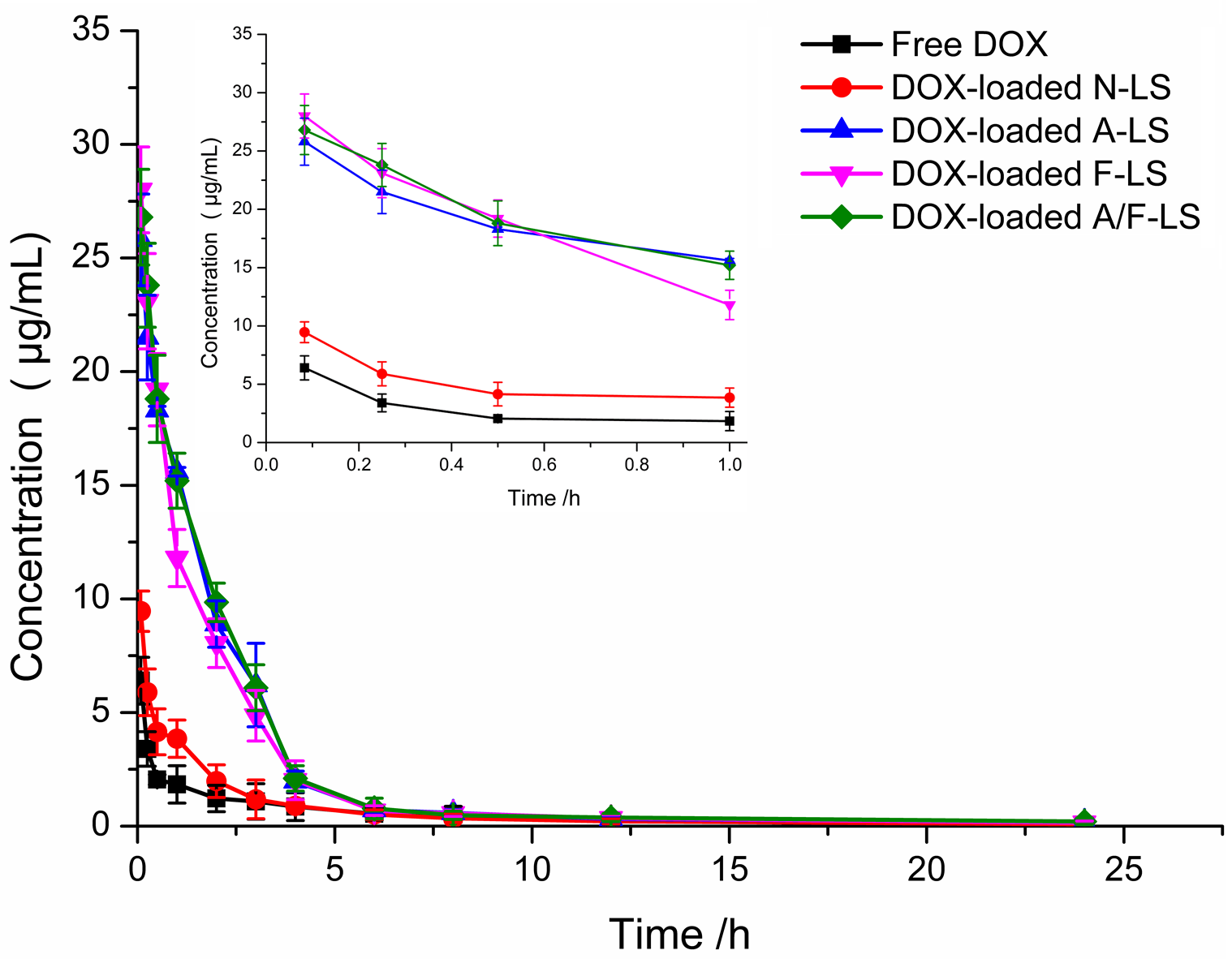
**

**Figure S3.** DOX concentration-time profile following intravenous administration of free DOX and various DOX-loaded liposoems in SD rats at the DOX dose of 5 mg/kg, respectively. The data are presented as the means ± SD (n = 6).
